# Supplementary material for: The Candida glabrata Upc2A transcription factor is a global regulator of antifungal drug resistance pathways
Source: PLoS Genet. 2021 Sep 30;17(9):e1009582. doi: 10.1371/journal.pgen.1009582 (PMC8509923; doi:10.1371/journal.pgen.1009582)
Supplement: Attachment — Submitted filename: Response-2_9-16-21.docx [file pgen.1009582.s006.docx]

Reviewer #1: The authors have done a fantastic job; the presentation now brings out the significance of the findings very effectively.
There are some minor details for the authors' consideration that I may have missed last time:
1. Lines 24-25. I believe that "which" is more appropriate than "that." I would say, "The most commonly used antifungal drugs are the azole compounds, which interfere with biosynthesis of the fungal-specific sterol: ergosterol." I could not easily find the relevant Strunk and White advice, but the principle is summarized well in this posting from Purdue University:
<https://owl.purdue.edu/owl/general_writing/grammar/that_vs_which.html>
Yes, I know that there are folks who dispute this advice. It's up to you in the end, of course.

**Response:** Changed as requested.

2. Line 238 and Figure 3. Just asking: are you sure that you want "WTF" in your publication? Another reviewer mentioned this issue previously and you responded that no other reviewer had mentioned it. But the fact that I missed it last time was just due to distraction, laziness, or any of the seven deadly sins.

**Response:** As our only intention here was to indicate wild-type cells treated with fluconazole, we are comfortable with this abbreviation.

3. Line 345. This line looks like a subheading but it is not indicated in bold as the others are.

**Response:** This is a second level subheading and is italicized to keep it separate from section dividers that are bolded.

Reviewer #2: Original Comment:

1) Several interesting observations are made with the ChIP-Seq. There are a large number of promoters (309) that are always bound by Upc2. There are also a large number of promoters that are bound in wild type upon fluconazole treatment and in the gain-of-function mutant (85). Yet this was a small fraction of the total number of promoters bound by Upc2 under FLC treatment. Was this small overlap expected? I would have thought for a true gain-of-function mutation, this overlap would be more substantial. Is there differences in the SRE promoter sequences under these different conditions?

Additional clarification and discussion is needed to explain why out of ~1,000 peaks identified in the ChIP-Seq that were bound by wild-type Upc2A in the presence of fluconazole, only 85 were detected in the gain-of-function mutant. Adding the statement included in the response to the review (or a similar sentiment) in the text itself would highlight this discrepancy to the reader:

“The large number of genes that only show binding in wild-type cells that are FLC-treated was surprising. It is important to note that these represent classes of genes that are not typically thought of as Upc2A-responsive, such as components of the translational machinery, and confirmation of the role of Upc2A in their expression awaits further experimentation.

**Response:** Edited as requested.

Original Comment:

2) For the RNA-seq data, the authors find 53 transcripts that require Upc2A for expression and that are bound by the regulator. These represent the direct targets. These should be more clearly described and highlighted in the text. Furthermore, I found it surprising that only 11 genes were significantly upregulated in the Upc2A gain-of-function (GOF) mutant. How do these compare with the 53 transcripts? Overall, my interpretation of this analysis is that Upc2A predominantly governs genes involved in the biosynthesis of ergosterol. The conclusion the Upc2A regulates other diverse sets of genes is likely largely indirect.

Additional clarification is needed for the RNA-Seq analysis. The authors need to clearly articulate that only 53 of the transcripts identified (representing less than 3% of transcripts analyzed) are both bound by Upc2 and induced upon fluconazole treatment in an azole-dependent manner. The other groupings of transcripts described in this section likely represent indirect targets (i.e. the 64 genes that are fluconazole induced in a Upc2A-dependent manner but not bound) or are transcripts that could be induced by Upc2A as well as other transcriptional regulators (i.e. the 274 genes that are fluconazole induced regardless of the presence or absence of Upc2, yet are also bound by Upc2A). A careful revision of this section is needed not only clearly state the conclusions of these findings, but also to walk the reader through the data in a logical manner.

**Response:** This information is presented in the two next paragraphs following the data mentioned by the reviewer.

Original Comment:

3) Ergosterol biosynthesis genes (ie ERG11) were shown to be exclusively bound by Upc2A (Fig. 2D) as opposed to being bound by both Upc2A and Pdr1 (ie CDR1). Therefore, it was puzzling in Fig. 5 when the authors introduced a mutant SRE sequence into the promoter regions of CDR1 and PDR1 but not ERG11. I would like to see the impact of mutating the SRE on the expression of a gene that is not also regulated by another key transcriptional regulator. How does mutating the ERG11 SRE impact fluconazole resistance? If there was a reason this was only performed in with ERG1 and not ERG11, this should be explained in text.

Thank you for adding this clarification. While I would still like to see the impact of mutating the SRE in the promoter of a gene ONLY regulated by Upc2A, I appreciate the need for a strong signal and am satisfied with at least including the rationale in the txt.

Original Comment:

**Response:** None required here.

4) Similarly, for assessing the role of Upc2A in anaerobic growth, I would like to see how mutating the ERG11 SRE impacts the induction of ERG11 in aerobic versus anaerobic conditions.

No longer applicable due to removal of data.

**Response:** None required here.

Original Comment:

5) From what I can tell, the only experiment that specifies number of replicates performed in the analysis is for the western blots. Please specify how many times the other experiments were performed. (Be specific regarding biological or technical analyses). This is especially important for qRT-PCR for which some of the “significant” changes are minimal.

Thank you for adding the details for the qRT-PCR experiments. However, this detail should be included for all other assays as well (spottings, B-galactosidase assay, DNaseI protection assay etc).

**Response:** All these experiments are repeated at least twice with a minimum of two technical and biological replicates.

Original Comment:

6) Overall the authors should revise the manuscript to ensure language and background information is as clear as possible. For example, line 143-146 should specify that it replaced the native UPC2A and line 191 should explain the p0 cells are C. glabrata without mitochondria. Furthermore, there were several typos such as line 202, take out “be”; line 450, change “early” to “earlier”; and line 549, add “where” in between “from we”.

Thank you for making these editorial corrections. As also pointed out by reviewer 3, additional care should be taken in the final revision to ensure all grammar and spelling are correct, as well as to revise the manuscript for clarity.

**Response:** This manuscript will be copy edited by the journal after acceptance which will deal with errors of this type.

Minor Comments:

All have been addressed except:

Original Comment:

The description of the genomic analyses (ChIP-Seq and RNA-seq) was confusing at times. Refining the text to provide systematic and clear Description of the findings would be beneficial.

Insufficient clarification/editorial revision was conducted for ChIP-Seq/RNA-seq sections. While the addition of sub-headings was helpful, this alone is not sufficient to provide the reader with extra clarity for these results. Specifically, for the RNA-seq only a few short sentences were added.

**Response:** I have revised this section again for clarity. No other reviewer mentioned an issue with the clarity of the revisions already provided.

Reviewer #3: None

Large datasets are available as a GEO reference Series: GSE182516. This has been included in the revised manuscript.
